# Therapeutic efficacies of artemether-lumefantrine and dihydroartemisinin-piperaquine for the treatment of uncomplicated *Plasmodium falciparum* and chloroquine and dihydroartemisinin-piperaquine for uncomplicated *Plasmodium vivax* infection in Ethiopia

**DOI:** 10.1186/s12936-022-04350-z

**Published:** 2022-12-01

**Authors:** Ashenafi Assefa, Hussein Mohammed, Anjoli Anand, Adugna Abera, Heven Sime, Anna A. Minta, Mekonnen Tadesse, Yehualashet Tadesse, Samuel Girma, Worku Bekele, Kebede Etana, Bereket Hailegiorgis Alemayehu, Hiwot Teka, Dereje Dilu, Mebrahtom Haile, Hiwot Solomon, Leah F. Moriarty, Zhiyong Zhou, Samaly Souza Svigel, Bryan Ezema, Geremew Tasew, Adugna Woyessa, Jimee Hwang, Matthew Murphy

**Affiliations:** 1 Ethiopian Public Health Institute, Addis Ababa, Ethiopia; 2 Institute for Global Health and Infectious Diseases, University of North Carolina at Chapel Hill, Chapel Hill, NC USA; 3 Epidemic Intelligence Service, U.S. Centers for Disease Control and Prevention, Atlanta, GA USA; 4 Malaria Branch, U.S. Centers for Disease Control and Prevention, Atlanta, GA USA; 5 ICAP at Columbia University, Addis Ababa, Ethiopia; 6 U.S. President’s Malaria Initiative, USA Agency for International Development, Addis Ababa, Ethiopia; 7 World Health Organization, Addis Ababa, Ethiopia; 8 Ethiopian Federal Ministry of Health, Addis Ababa, Ethiopia; 9 U.S. President’s Malaria Initiative, Malaria Branch, US Centers for Disease Control and Prevention, Atlanta, GA USA

## Abstract

**Background:**

Routine monitoring of anti-malarial drugs is recommended for early detection of drug resistance and to inform national malaria treatment guidelines. In Ethiopia, the national treatment guidelines employ a species-specific approach. Artemether-lumefantrine (AL) and chloroquine (CQ) are the first-line schizonticidal treatments for *Plasmodium falciparum* and *Plasmodium vivax*, respectively. The National Malaria Control and Elimination Programme in Ethiopia is considering dihydroartemisinin-piperaquine (DHA/PPQ) as an alternative regimen for *P. falciparum* and *P. vivax*.

**Methods:**

The study assessed the clinical and parasitological efficacy of AL, CQ, and DHA/PPQ in four arms. Patients over 6 months and less than 18 years of age with uncomplicated malaria mono-infection were recruited and allocated to AL against *P. falciparum* and CQ against *P. vivax*. Patients 18 years or older with uncomplicated malaria mono-infection were recruited and randomized to AL or dihydroartemisinin-piperaquine (DHA/PPQ) against *P. falciparum* and CQ or DHA/PPQ for *P. vivax*. Patients were followed up for 28 (for CQ and AL) or 42 days (for DHA/PPQ) according to the WHO recommendations. Polymerase chain reaction (PCR)-corrected and uncorrected estimates were analysed by Kaplan Meier survival analysis and per protocol methods.

**Results:**

A total of 379 patients were enroled in four arms (n = 106, AL-*P. falciparum*; n = 75, DHA/PPQ- *P. falciparum*; n = 142, CQ-*P. vivax*; n = 56, DHA/PPQ-*P. vivax*). High PCR-corrected adequate clinical and parasitological response (ACPR) rates were observed at the primary end points of 28 days for AL and CQ and 42 days for DHA/PPQ. ACPR rates were 100% in AL-Pf (95% CI: 96–100), 98% in CQ-*P. vivax* (95% CI: 95–100) at 28 days, and 100% in the DHA/PPQ arms for both *P. falciparum* and *P. vivax* at 42 days. For secondary endpoints, by day three 99% of AL-*P. falciparum* patients (n = 101) cleared parasites and 100% were afebrile. For all other arms, 100% of patients cleared parasites and were afebrile by day three*.* No serious adverse events were reported.

**Conclusion:**

This study demonstrated high therapeutic efficacy for the anti-malarial drugs currently used by the malaria control programme in Ethiopia and provides information on the efficacy of DHA/PPQ for the treatment of *P. falciparum* and P. vivax as an alternative option.

**Supplementary Information:**

The online version contains supplementary material available at 10.1186/s12936-022-04350-z.

## Background

Malaria remains a disease of significant public health importance in Ethiopia despite the gains made through recent malaria control efforts. In 2020, the World Health Organization (WHO) estimated approximately 4.2 million malaria cases in Ethiopia with *Plasmodium falciparum* accounting for 77% of the confirmed cases [1]. According to the Malaria Programme Review conducted by the WHO in April 2020, deaths due to malaria decreased 67%, from 9/100,000 to 3/100,000 population at risk, and the annual parasite incidence decreased 37%, from 19/1,000 to 12/1,000 population, between 2016 and 2019 [2]. Building on this progress, Ethiopia aims to eliminate malaria by 2030 [3]. Prompt case management with efficacious drugs plays a pivotal role in malaria control and elimination efforts [4]. Artemether-lumefantrine (AL) is the current first-line anti-malarial medication for uncomplicated *P. falciparum*, and chloroquine (CQ) remains the first-line treatment for *P vivax* in Ethiopia. The second-line treatment for *P. falciparum* uncomplicated malaria is oral quinine and for uncomplicated *P. vivax* malaria is AL [5]. The Ethiopian Ministry of Health (MOH) is considering dihydroartemisinin-piperaquine (DHA/PPQ) as an alternative regimen and seeks to generate safety and efficacy data in Ethiopia prior to any policy changes.

The Ethiopian Public Health Institute (EPHI), collaborating with local and international partners (WHO and U.S. President’s Malaria Initiative (PMI)), has been monitoring the therapeutic efficacy of anti-malarial drugs that may be used for malaria management in the country. With the exception of data from a location in Arbaminch in 2008, where efficacy was reported to be 92.5% (Personal communication, Moges Kassa), AL efficacy remained greater than 95% against uncomplicated *P. falciparum* throughout the country since the beginning of its use in 2004 [6–10]. However, the evidence of resistance to artemisinins in Southeast Asia and emergence of an artemisinin-resistance-associated mutation in Rwanda underscores the need for continued surveillance [11, 12]. Chloroquine-resistant *P. vivax* has remained rare in Africa. In Ethiopia, CQ efficacy remains above 95% although sporadic reports of CQ failure suggest emerging resistance [8, 13–20]. There are limited data in Ethiopia on the efficacy of other artemisinin-based combinations.

Although DHA/PPQ is a WHO-recommended treatment for malaria (regardless of species) and the first-line therapy in many countries in Asia and Africa, its efficacy has not been evaluated in Ethiopia to date. It has been demonstrated to be highly effective against both *P. falciparum* and *P. vivax* and well-tolerated in Africa and Asia [21–25]. Gastrointestinal distress and dizziness are the most commonly reported adverse events [23]. QT prolongation without clinical abnormalities or cardiac toxicity has also been noted [4].

This study reports the therapeutic efficacy of AL or DHA/PPQ against uncomplicated *P. falciparum* and CQ or DHA/PPQ against uncomplicated *P. vivax* to provide on-going, evidence-based recommendations for the national malaria treatment guidelines.

## Methods

The study evaluated adequate clinical and parasitological responses (ACPR) to standard therapeutic doses of AL or DHA/PPQ in patients with uncomplicated *P. falciparum* and CQ or DHA/PPQ in patients with uncomplicated *P. vivax.*

### Study area and population

**Fig. 1:**
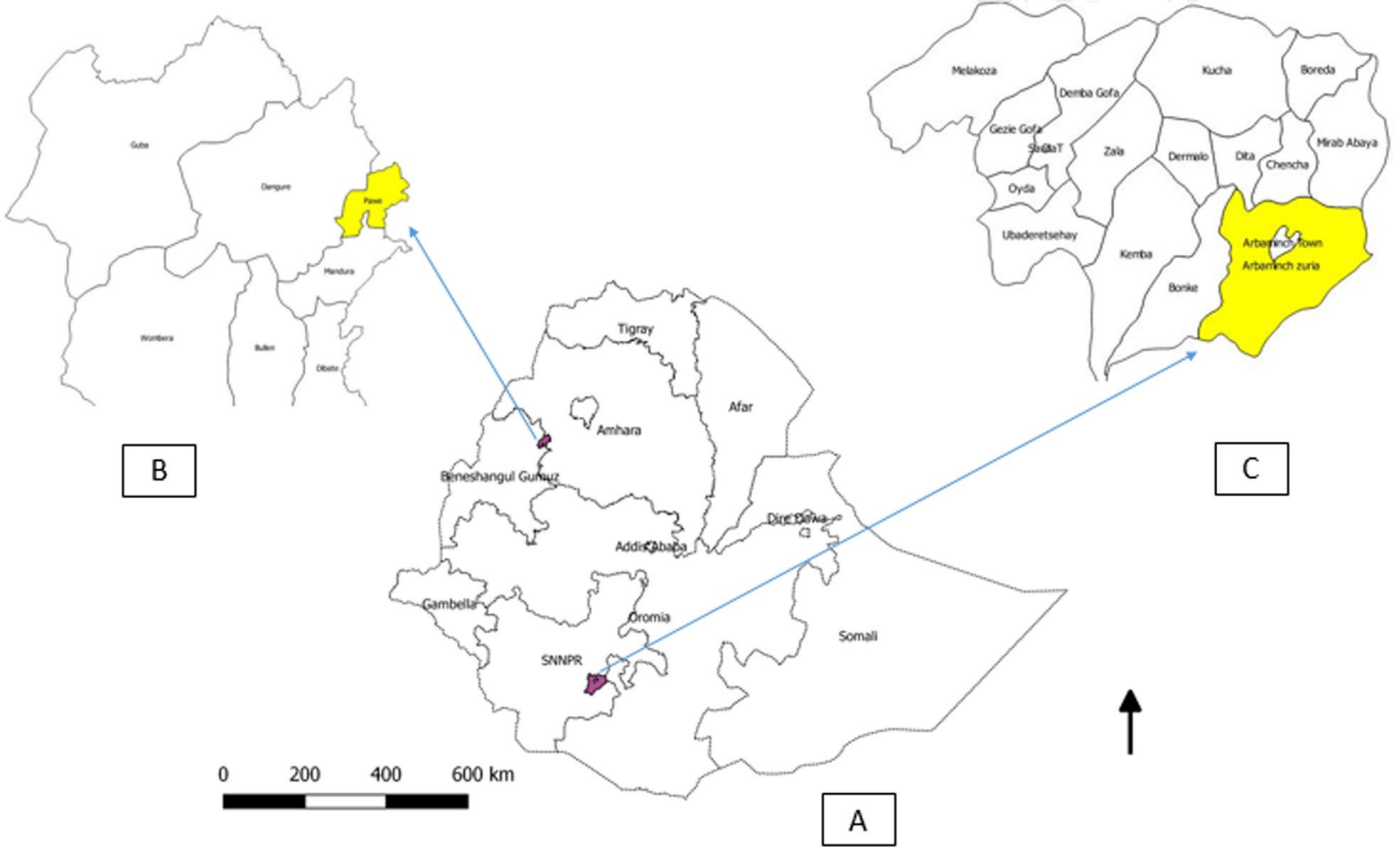
Map of the study area. **A** Map of Ethiopia with study areas in Benishangul-Gumuz and Southern Nations and Nationalities Peoples’ (SNNP) Regions noted in purple. **B** Location of Pawe in Metekel Zone, Benishangul-Gumuz Region. **C** Location of Arbaminch in Gamu-Gofa Zone, SNNP Region. *NB* B and C are not to scale)

The study was conducted in two sentinel sites in Ethiopia: (1) Felegeselam Health Centre, Pawe, Metekel Zone, Benishangul Gumuz Region and (2) Arbaminch Health Centre, Gamu-Gofa Zone, Southern Nations and Nationalities Peoples’ (SNNP) Region (Fig. 1). Felegeselam Health Centre is located 589 km from Addis Ababa in northwestern Ethiopia. Arbaminch Health Centre is located about 500 km southwest of Addis Ababa. Therapeutic efficacy studies have been conducted in these areas previously: 2008 and 2011 in Arbaminch, and 2010 and 2013 in Pawe.

The study areas have moderate malaria transmission and malaria affects all age groups. *Plasmodium falciparum* and *P. vivax co*-exist at these sites, with *P. falciparum* being the predominant species. *Anopheles arabiensis* is the primary malaria vector. The study was conducted September–December 2017 during the major malaria transmission season.

### Study design and participants

The study was designed as an open label, four arm trial conducted in two sites (*P. falciparum-*AL, *P. falciparum*-DHA/PPQ, *P. vivax*-CQ and *P. vivax*-DHA/PPQ arms). The study was based on the WHO recommendations for designing surveillance studies on anti-malarial drug efficacy [26]. Patients presenting to the outpatient department with mono-infection for *P. falciparum* or *P. vivax* were enroled in the study. *Plasmodium falciparum*-infected patients 18 years of age and above were randomized to the AL or DHA/PPQ arms. All patients with *P. falciparum* older than six months and younger than 18 years were enroled in the AL arm. Similarly, patients with *P. vivax* 18 years of age and above were randomized to the CQ or DHA/PPQ arms. All patients older than six months and younger than 18 years were enroled in the CQ arm. Enrolment to DHA/PPQ arms were limited to adults 18 years of age and above as per the guidance of the Food, Medicine and Health Care Administration and Control Authority of Ethiopia (FMHACA).

The WHO recommendations for inclusion and exclusion were followed with the notable addition of weight ≥ 5 kg based on WHO dosing recommendations, lowering of the minimal enroling *P. falciparum* asexual parasite count from 1,000 to 500 parasites/µL based on local transmission, and the addition of a residency restriction of within 20 km from the enroling health facility to facilitate visiting the patient if needed [26]. All participants or their guardian/caregiver agreed to the finger prick sampling and provided written informed consent/assent.

### Coordination and quality control

The study was coordinated and implemented by EPHI and ICAP at Columbia University in Addis Ababa, Ethiopia. A three-day training of trainers (TOT) was conducted to review the study protocol for the central study team. The site teams comprised of six people per site: two clinicians, two laboratory technologists, a porter/tracer, and a supervisor. The central team provided on-site training and supervision for the first two weeks of study enrolment. The site teams received additional regular supportive supervision throughout the study period.

### Treatment and follow up

Patients with *P. falciparum* enroled in the study were treated with either AL or DHA/PPQ (if ≥ 18 years of age), and patients with *P. vivax* were treated with either CQ or DHA/PPQ (if ≥ 18 years of age). Artemether-lumefantrine (20 mg of artemether and 120 mg of lumefantrine; Novartis Pharmaceuticals Corporation, New York, NY, US) was administered twice daily for three days, and DHA/PPQ (40 mg DHA and 640 mg PPQ), Duo-CotecxinR, Holley-Cotec Pharmaceuticals, China) was administered once daily for 3 days according to the manufacturers’ recommendations. CQ (Micro Labs Limited, Tamil Nadu, India) was prescribed according to national treatment guidelines at 25 mg base/kg over 3 days (10 mg base/kg on days 0 and 1, and 5 mg base/kg on day 2). All study drugs were provided by the WHO Global Malaria Programme. All DHA/PPQ and CQ treatment doses were given under the direct supervision of study clinicians and study team members, whereas for AL only the morning dose was supervised, the evening dose was taken at home and patients were asked if they took the drug as instructed before administering the next dose. Patients were encouraged to eat fatty foods and expected to report completion of the second, evening AL dose.

All patients were observed for adverse reactions or vomiting for 60 min following treatment administration. Patients vomiting their medication within the first 30 min received a repeat full dose; patients vomiting within 30–60 min received half the original dose. Patients with *P. vivax* mono- or mixed infection during enrolment or follow-up were offered treatment with 14 days of primaquine (0.25 mg/kg) (Sanofi-Aventis, Bridgewater, NJ, US) as per the national treatment guideline for radical cure at the end of the follow-up period or upon reaching a study endpoint.

### Clinical procedures

At enrolment, patients completed a medical examination and questionnaire, and a capillary blood sample was collected for blood film examination and haemoglobin measurement. In addition, three 50-μl capillary blood spots were stored on filter papers (Whatman 903 and Whatman 3; GE Healthcare Biosciences, Westborough, MA, US).

Patients were asked to return for routine assessment, follow-up medical exam, and blood film examination on days 1, 2, 3, 7, 14, 21, 28, 35, and 42. For the primary efficacy outcome, AL and CQ were assessed to day 28 and DHA/PPQ to day 42 as recommended by WHO. Polymerase chain reaction (PCR) correction was only done to day 28 for AL and CQ arms. Patients were also asked to return to the clinic if they had signs or symptoms consistent with malaria or any adverse events on non-scheduled follow up days. Adverse events and concomitant medications were recorded at every visit, and repeat haemoglobin concentration was measured on days 0, 14, 28 and 42.

### Classification of treatment outcome

According to WHO recommendations, treatment responses were classified as early treatment failure (ETF), late clinical failure (LCF), late parasitological failure (LPF), or ACPR for *P. falciparum* and *P. vivax* [26].

#### Early treatment failure

Development of danger signs or severe malaria on day 1, 2 or 3, in the presence of parasitaemia; parasitaemia on day 2 higher than on day 0, irrespective of axillary temperature; parasitaemia on day 3 with axillary temperature ≥ 37.5 °C; and parasitaemia on day 3 ≥ 25% of count on day 0.

#### Late clinical failure

Development of danger signs or severe malaria in the presence of parasitaemia on any day between day 4 and day 28 in patients who did not previously meet any of the criteria of early treatment failure; and presence of parasitaemia on any day between day 4 and day 28 with axillary temperature ≥ 37.5 °C in patients who did not previously meet any of the criteria of early treatment failure.

#### Late parasitological failure

Presence of parasitaemia on any day between day 7 and day 28 with axillary temperature < 37.5 °C in patients who did not previously meet any of the criteria of early treatment failure or late clinical failure.

#### Adequate clinical and parasitological response

Absence of parasitaemia on day 28 or 42, irrespective of axillary temperature, in patients who did not previously meet any of the criteria of ETF, LCF or LPF.

The primary endpoints were PCR-corrected ACPR on day 28 for AL and CQ and day 42 for DHA/PPQ. Other outcomes were loss to follow-up and withdrawals which included protocol violation, withdrawal of consent/assent, interference (taking another drug with anti-malarial activity), and failure to complete study treatment (including persistent vomiting, concomitant disease, mixed infection).

### Laboratory procedures

#### Microscopic examination

Blood samples collected by finger prick from febrile outpatients were stained by 10% Giemsa for 10–15 min for initial screening. Blood films were examined by two microscopists and all slides were read independently. When the patient was enroled, and at subsequent follow-up visits, thick and thin blood smears were prepared on a single slide for parasite detection and species identification. Slides were stained by 3% Giemsa for 45 min, and then parasites were counted on thick films as the number of asexual parasites per 200 white blood cells (or per 500, if the count was < 10 parasites/200 white blood cells). A smear was declared negative if no parasites were seen after 1000 white blood cells were counted. The presence of gametocytes at enrolment or during follow-up was recorded. Asexual parasite density per microlitre (μL) was calculated on the assumption of 8000 leucocytes per μL blood. All collected slides were cross-checked by WHO-certified microscopists (Adama Malaria Control Centre, Ethiopia) after the study. If the two parasitaemia readings were in agreement (difference in parasite densities < 50%), the average results were recorded. If the two counts were discordant in terms of parasite species or density by > 50%, then a third, independent microscopist re-examined the blood slides. For parasite positivity and species, two concordant results were considered the final result, while for parasite density, the average of the two closest estimates of parasitaemia was considered final.

#### Haemoglobin

Haemoglobin was measured from finger prick blood samples using a portable spectrophotometer (HemoCue, Ängelholm, Sweden) on days 0, 14, 28, and 42.

#### Parasite genotyping

In order to differentiate recrudescence from re-infection, blood samples were collected on filter paper from a finger-prick on day 0 and on the day of parasite recurrence (day 7 onwards) for genotyping. Specimens were dried, stored in individual plastic bags with desiccants and protected from light, humidity, and extreme temperatures. The samples were genotyped at the U.S. Centers for Disease Control and Prevention (CDC) in Atlanta, GA, USA. Each dried blood spot was punched with a sterile puncher and DNA from the punched spots were extracted using QIAamp® DNA Mini Kit (QIAGEN, Valencia, CA). For *P. falciparum* positive samples, seven neutral microsatellite markers (C2M34-313 on chromosome 2, C2M69-383 on chromosome 3, Poly-α on chromosome 4, TA1 and TAI09 on chromosome 6, 2490 on chromosome 10, and PfPK2 on chromosome 12) were analyzed as previously published [27]. Microsatellite allele size and peak height (above 200 fluorescent units) were scored by GeneMarker v3.0.0 (SoftGenetics, PA, USA) from all seven markers. For *P. vivax* samples, seven microsatellite markers (3.502 on chromosome 3, MS2 and MS038 on chromosome 6, 10.29 on chromosome 10, 11.162 and MS6 on chromosome 11, and 12.335 on chromosome 12) were genotyped using published protocols [27, 28]. Background allele frequencies were determined from day 0 of a randomly selected 20% of samples from participants classified as ACPR. A previously described Bayesian statistical algorithm was used to assign a posterior probability of recrudescence to each case of recurrent parasitaemia [29] (Additional file 1).

### Molecular markers

All samples of *P. falciparum* recurrence, in addition to 20% of randomly selected day zero samples, were analysed for polymorphisms in the p*fk13* propeller domain and *pfmdr1* by performing Sanger sequencing [30]*.* The *pfK13* domain from codon positions 389–649 was assessed for presence of mutations known to be associated with artemisinin resistance as recommended by the WHO (N458Y, Y493H, R539T, I543T, C580Y, F446I, M476I, R561H, P553L) using a previously published method [30–32]. Five mutations in the *pfmdr1* gene that are associated with resistance to different anti-malarial drugs were analysed at codons N86Y, Y184F, S1034C, N1042D, D1246Y as reported previously [32].

### Sample size

Assuming an ACPR rate of 95% and 95% confidence interval and 5% precision, a total of 73 patients per arm were calculated. Factoring in 20% loss to follow-up and withdrawals, 88 patients per each arm for a total of 352 patients across the four arms were targeted.

### Statistical analysis

SAS 9.3 (Cary, NC) and the WHO Excel-based data entry and analysis tool were used for analysis [33]. Data were analysed as per-protocol and Kaplan–Meier (survival) analysis methods. Patients who were withdrawn from the study or lost to follow-up were excluded from the per-protocol analysis; patients with PCR-confirmed reinfection were excluded from the PCR-corrected per-protocol analysis. For the survival analysis, patients who were lost to follow-up or withdrawn were censored on the last day of follow-up according to the timetable. Reinfections were censored on the last day of follow up in the PCR-corrected survival analysis.

### Ethical considerations

The study protocol was approved by the Ethiopian Public Health Institute (EPHI), the National Ethical Committee in Ethiopia (3.10/171/2016) and FMHACA. In addition, the study was reviewed and approved by the Institutional Review Boards (IRBs) of Columbia University and the U.S. CDC (#6892). Written consent and/or assent was obtained from study participants or their guardians.

## Results

**Fig. 2:**
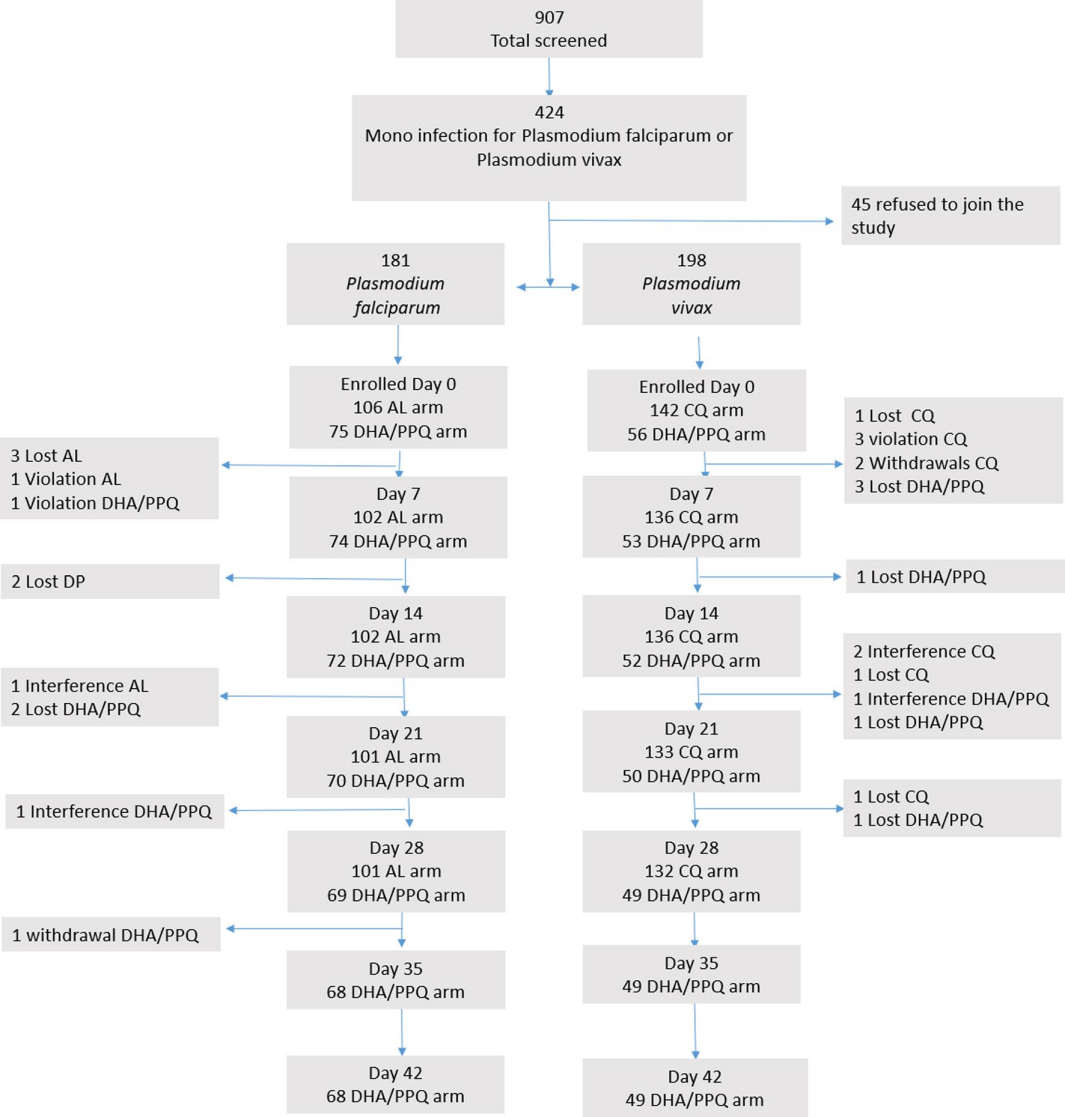
Flow chart of the study enrolment and follow-up. *Lost* lost to follow up, *Violation* protocol violation, *AL* Artemether-Lumefantrine, *DHA/PPQ* Dihydroartemisinin-Piperaquine, *CQ* Chloroquine

A total of 10,903 febrile patients presented to Felegeselam and Arbaminch Health Centres during the study period. Of these, 907 patients were screened in the laboratory. Four hundred twenty-four patients mono-infected with either *P. falciparum* or *P. vivax* fulfilled the inclusion criteria*.* As the number of cases per each site was not sufficient for per site analysis, data was pooled from the two study sites (supplementary tables 1–3 are included to show per site analysis). One hundred and eighty-one *P. falciparum*-infected patients were enroled in AL (n = 106) and DHA/PPQ (n = 75) arms. One hundred and ninety-eight *P. vivax*-infected patients were enroled in the CQ (n = 142) and DHA/PPQ (n = 56) arms. Most *P. falciparum* infected patients were enroled in Arbaminch (n = 156) and most P. vivax patients were enroled at Pawe (n = 144) health centres. A total of 101 (95.3%) *P. falciparum* patients in the AL arm and 132 (93.0%) patients in the *P. vivax*-CQ arm reached the 28-day primary endpoint; 68 (90.7%) *P. falciparum* and 49 (87.5%) *P. vivax* patients in the DHA/PPQ arms completed the 42 days of follow up (Fig. 2). The study was terminated at the end of the transmission season despite not reaching the targeted sample size for each site.

The median parasitaemia at day zero were 18,255 for AL, and 8,755 for DHA/PPQ arms for *P. falciparum* and 10,051 for CQ and 5,373 for DHA/PPQ arms for *P. vivax*. The median age was 15 years (range: 1–57) in the *P. falciparum-*AL arm and 25 years (18–65) in the *P. falciparum-*DHA/PPQ arm. The median age was 14 (1–70) in the *P. vivax-*CQ arm and 23 (18–70) in the P*. vivax*-DHA/PPQ arm. Most of the study participants were male (56–71%) in all arms. Forty two percent of patients enroled had gametes: 77% (152/198) *P. vivax*-infected and 5% (9/181) *P. falciparum*-infected patients had gametes. The median day 0 gametocyte density for *P. falciparum* was zero per µl for both arms but with a range of 0–3,520 in the DHA/PPQ arm; for *P. vivax*, the median was 401 per µl (range: 0–14,023) in the CQ arm and 320 per µl (range: 0–10,000) in the DHA/PPQ arm. Median day zero haemoglobin levels were similar across the four arms (Table 1).

**Table 1:** Study profile and characteristics of patients enrolled in Felegeselam and Arbaminch Health Centers, Ethiopia, 2017

	*P. falciparum*		*P. vivax*	
	AL n = 106	DHA/PPQ n = 75	CQ n = 142	DHA/PPQ n = 56
Median age, years (range)	15 (1–57)	25 (18–65)	14 (1–70)	23 (18–70)
Age groups, years (n)				
6 months̵̵̵– ≤ 5 years	2	n/a	17	n/a
> 5– < 18 years	75	n/a	82	n/a
≥ 18 years	29	75	43	56
Median weight, kg (range)	45 (8–79)	53 (39–71)	42 (9–78)	59 (41–106)
Percent male, %	66.0	70.7	56.3	64.3
Median day 0 parasitaemia, parasites/µl (range)	18,255 (520–97,326)	8755 (524–96,000)	10,051 (520–48,320)	5373 (737–30,320)
Median day 0 gametocyte density, gametocytes/µl (range)	0	0 (0–3520)	401 (0–14,023)	320 (0–10,000)
Median day 0 haemoglobin, g/dl (range)	13.3 (8.6–18.7)	13.3 (7.4–17.6)	12.8 (7.3–18.3)	14.3 (9.9–17.6)

*P* Plasmodium, *AL* artemether-lumefantrine, *DHA/PPQ* Dihydroartemisinin-piperaquine, *CQ* Chloroquine

The treatment outcomes for day 28 and 42 (limited to DHA/PPQ) are shown in Table 2. Using the per-protocol analysis, the 28-day follow-up ACPR for *P. falciparum* was 98% (95% CI: 93–100) for AL and 100% for DHA/PPQ. The 42-day follow-up ACPR for DHA/PPQ was 100%. Two failures in the AL arm were observed, one on day 21 and one on day 28. Both failures were confirmed to be re-infection by PCR, one with a 33% and one with 11% probability of recrudescence. The 28-day follow-up PCR-corrected ACPR for CQ against P*. vivax* was 98% (95% CI: 94–100), with the two PCR-corrected samples with probability of recrudescence above 99%. The 42-day follow-up ACPR for DHA/PPQ against *P. vivax* was 100% (95% CI 93–100). The Kaplan–Meier analysis is presented to show the censored estimates (Table 2) and microsatellite data are included as a supplement. Although not a primary outcome, 42-day follow up ACPR for CQ was 82% with Felgeselam Health Centre reporting 86.0% and Arbaminch 97.7% (data not shown).

**Table 2:** Treatment outcomes in patients with uncomplicated *Plasmodium falciparum* and *P. vivax* infections treated with AL and DHA/PPQ for *P. falciparum,* and CQ and DHA/PPQ for *P. vivax*, Ethiopia, 2017

	*P. falciparum*		*P. vivax*	
	AL n = 101	DHA/PPQ n = 68	CQ n = 132	DHA/PPQ n = 49
Late clinical failure	4	0	4	0
Late parasitological failure	2	0	18	0
Day of failure, 19–21	1	0	1	0
Day of failure, 22–28	1	0	2	0
Day of failure, 29–35	2	0	10	0
Day of failure, 36–42	2	0	9	0
Adequate clinical and parasitological response (% [95%CI])—day 28	99 (98% [93–100%])	68 (100% [95–100])	129 (98 [94–100%])	49 (100 [93–100%])
Kaplan–Meier estimate, uncorrected (95% CI)—day 28	98% (95–100%)	100%	98% (95–100%)	100%
Adequate clinical and parasitological response (%[95%CI])—day 42	–	68 (100% [95–100%])	–	49 (100% [93–100%])
Kaplan–Meier estimate, uncorrected (95% CI)—day 42	–	100%	–	100%
PCR-correction^a^				
Recrudescence	0	0	2	0
Reinfection	2	0	0	0
PCR-correction not available	0	0	1	0
Adequate clinical and parasitological response, PCR-corrected (% [95%CI])—day 28	99 (100% [96–100%])	–	129 (98% [95–100%]	–
Kaplan–Meier estimate, PCR-corrected (95% CI)—day 28	100%	–	99% (96–100%)	–

*AL* artemether-lumefantrine, *DHA/PPQ* dihydroartemisinin-piperaquine, *CQ* Chloroquine

^a^PCR correction only done to day 28 for AL and CQ arms

Table 3 presents data for secondary outcomes including day three parasite clearance. Asexual parasite clearance by day three was observed in all participants in all the arms by day three. One (1%) asexual parasite on the DHA/PPQ against *P. falciparum* arm and three (2%) asexual parasites on the CQ arm against P. vivax was observed on day two, that eventually cleared on day three. All patients cleared gametocytes. Two P. vivax-infected patients had gametocytes noted on day 28 in the CQ arm (Table 3). However, several patients in the CQ arm were observed to have gametes and gametocytes on day 35 (6%) and on day 42 (4%) (data not shown).

**Table 3:** Secondary outcomes in patients with uncomplicated *P. falciparum* and *P. vivax* infections treated with AL or DHA/PPQ for *P. falciparum,* and CQ or DHA/PPQ for *P. vivax*, Ethiopia, 2017

	*P. falciparum*		*P. vivax*	
	AL n = 102	DHA/PPQ n = 74	CQ n = 137	DHA/PPQ n = 56
Proportion afebrile, n (%)				
Day 1	74 (73%)	70 (95%)	130 (95%)	53 (95%)
Day 2	102 (100%)	74 (100%)	137 (100%)	56 (100%)
Day 3	101 (100%)	74 (100%)	137 (100%)	56(100%)
Proportion parasite-clear, asexual, n (%)				
Day 2	97 (96%)	74 (100%)	137 (100%)	56 (100%)
Day 3	100 (99%)	74 (100%)	137 (100%)	56 (100%)
Gametocyte carriage, n (%)				
Day 2	0 (0%)	1 (1%)	3 (2%)	0 (0%)
Day 3	0 (0%)	0 (0%)	0 (0%)	0 (0%)
Day 7	0 (0%)	0 (0%)	0 (0%)	0 (0%)
Day 14	0 (0%)	0 (0%)	0 (0%)	0 (0%)
Day 21	0 (0%)	0 (0%)	0 (0%)	0 (0%)
Day 28	0 (0%)	0 (0%)	2 (2%)	0 (0%)
Day 35	-	0 (0%)	–	0 (0%)
Day 42	-	0 (0%)	–	0 (0%)
Mean Hb concentration, g/dl				
Day 0	14.0	13.8	12.75	14.0
Day 14	12.5	12.7	13.0	13.6
Day 28	12.7	13.2	13.2	14.4
Day 42	-	14.3	–	14.5

*AL* artemether-lumefantrine, *DHA/PPQ* dihydroartemisinin-piperaquine, *CQ* Chloroquine

In all four arms, the study participants were afebrile by day two. In both DHA/PPQ arms, average haemoglobin levels initially decreased but then recovered by day 42 to levels higher than those on day zero (Table 3). No serious adverse events were observed and no cardiovascular-related complaints were reported.

### Resistance markers

A total of 50 Day 0 samples, 30 from the *P. falciparum*-AL arm and 20 from the *P. falciparum*-DHA/PPQ arm were sequenced for polymorphisms in the *pfk13* and *pfmdr1* genes. In the *pfk13* gene, nine haplotypes associated with artemisinin resistance were assessed (N456Y, Y493H, R539T, I543T, C580Y, F446I, M476I, R561H, P553L), and no resistance-related mutants were observed. One *pfk13* non-synonymous mutation, E433D was observed in a day zero sample from Pawe. In the *pfmdr1* gene, nine haplotypes were assessed, and the following mutations were observed: in one sample N86Y (2%), in two samples Y184Y/F (4%), in 35 samples Y184F (73%), and in one sample S1034N (2%). In total, 24/30 (80%) samples showed *pfmdr1* mutant haplotypes in the *P. falciparum*-AL arm and 15/20 (75%) samples in the *P. falciparum*-DHA/PPQ arm. Prevalence and polymorphism of resistance markers are shown in Table 4.

**Table 4:** Prevalence of *Pfk13* and *Pfmdr1* polymorphisms in day 0 samples (all samples and only those that were reinfected) and day of failure samples (recurrent infections), Ethiopia, 2017

Polymorphism	Background Prevalence (all D0 samples) N = 48		Reinfection (D0) N = 2		Reinfection (DF) N = 2	
	N	Percent (%)	n	Percent (%)	n	Percent (%)
*Pfk13*						
Samples sequenced	48	100	2	100	2	100
Wild type (no mutations detected)	48	100	2	100	2	100
*Pfmdr1*†						
N86	47	98	2	100	2	100
86**Y**	1	2	0	0	0	0
Y184	11	23	0	0	0	0
184**Y/F**	2	4	0	0	0	0
184**F**	35	73	2	100	2	100
S1034	47	98	2	100	2	100
1034**N**	1	2	0	0	0	0
D1246	48	100	2	100	2	100
1246**D/Y**	0	0	0	0	0	0
1246**Y**	0	0	0	0	0	0
Samples sequenced for haplotype analysis						
NYD	13	27	0	0	0	0
YFD	1	2	0	0	0	0
NFD	36	75	2	100	2	100
NFY	0	0	0	0	0	0
NYY	0	0	0	0	0	0
YYD	0	0	0	0	0	0
YFY	0	0	0	0	0	0
YYY	0	0	0	0	0	0

*D0* day zero, *DF* day of failure; Bold letter denotes an encoded amino acid change; No mutations found at *Pfmdr1* codon 1042 and 1246;

^†^Totals may not sum due to mixed infections

## Discussion

The current study reported the therapeutic efficacy of AL and DHA/PPQ against uncomplicated *P. falciparum,* and CQ and DHA/PPQ against uncomplicated *P. vivax.* All the study drugs showed high efficacy within their respective follow-up period, demonstrating that the first-line species-specific malaria treatments are efficacious in the study sites of Ethiopia. The PCR-corrected per-protocol analysis demonstrated 100% efficacy of AL against *P. falciparum* for the 28-day follow-up outcome and a 100% efficacy of DHA/PPQ against *P. falciparum* in the 42-day follow-up outcome. In a similar fashion, the PCR-corrected per-protocol analysis demonstrated 98% efficacy of CQ against *P. vivax* for the 28-day follow-up outcome, and 100% efficacy of DHA/PPQ for the 42-day follow-up outcome.

The high efficacy reported for AL and CQ was consistent with previous studies in Ethiopia. Studies conducted since 2006 by EPHI and others have shown a high efficacy of AL against *P. falciparum*, confirming the drug remains well-tolerated and effective for the intended use in Ethiopia [4, 5, 8–10, 15, 19, 34, 35]. The exception was a non-peer reviewed study in 2010 that reported 7.2% failure (92.8% uncorrected ACPR) of AL at the Shele Health Centre, Arbaminch Zuria in Southwest Ethiopia which is located close to the Arbaminch Health Centre in the current study (Moges Kassa personal communication). Although the lower efficacy result from this site with anecdotal reports of wide herbal artemisinin use was concerning, this study along with others have shown high efficacy of ACTs. DHA/PPQ has not been used for malaria treatment in the public sector in Ethiopia. However, the findings are consistent with previous reports of high efficacy for both *P. falciparum* and P. vivax from elsewhere and the extended protection to day 42 attributed to piperaquine’s longer half-life [21–25].

Asexual and sexual parasites and fever were cleared by day three in all arms, reaffirming the sensitivity of the parasites circulating in the population to the respective drugs, especially the artesiminin component [26]. Gametocytes were cleared by day three in all four arms; however, two patients with gametocytes were observed on day 28 in the P. vivax-CQ arm. No presence of gametocytes was observed in either of the *P. falciparum* and P. vivax DHA/PPQ arms up to 42-day follow-up by microscopy, contrasting with other data indicating that piperaquine may encourage gametocyte production when provided as mono-therapy [36]. As low density parasites may not be detected by microscopy; a sensitive quantitative nucleic acid detection method may be required to better understand parasite dynamics post-treatment [37–39]. The hematological profile of the participants in the DHA/PPQ arms showed a general initial decrease in haemoglobin levels with recovery by day 42 to levels similar to day 0 values, which is consistent with prior studies in Ethiopia as well as the findings of a systematic review of DHA/PPQ treatment for P. vivax [8, 40]. All study drugs were shown to be generally well-tolerated with no serious adverse events observed.

Among the 50 *P. falciparum* samples sequenced for the *PfK13* gene, none had a mutation associated with artemisinin resistance. These results were in contrast with previous studies that showed the presence of the k13 markers in Ethiopia and elsewhere in Africa [12, 41, 42]. None of the isolates reported in Africa were related to treatment failure, except the R561H haplotype reported from Rwanda, which was associated with confirmed delayed parasite clearance [12, 42]. R561H was not detected in the current study. This study reported a considerable number of the samples (36/50) with the NFD haplotype. Certain *Pfmdr1* haplotypes have been associated with CQ, mefloquine, quinine, other quinolones, and/or artemisinin resistance [26, 31]. A case study based on an Italian tourist who travelled to Africa, including Ethiopia, reported treatment failure after DHA/PPQ with presence of *pfmdr1* markers. The markers were assumed to be related with piperaquine, suggesting the need for continuous molecular surveillance for anti-malarial resistance [43].

Chloroquine resistance against P. vivax has been reported in Ethiopia and in Southeast Asia [15, 34, 42]. Despite the six decades of use in Ethiopia, the 98% ACPR at day 28 and the high parasite clearance rates on days 2 and 3 for CQ are encouraging [5]. The lower and site-specific differences noted on the 42-day ACPR is likely a reflection of different local transmission levels as these recurrences are due to relapses or reinfection. The addition of primaquine for radical cure which is now included in the revised national malaria treatment guideline of Ethiopia will not only address relapse prevention but should further enhance the schizonticidal efficacy of CQ [8, 44].

The high efficacy of DHA/PPQ has been reported in numerous African countries and Southeast Asia. Although only studied in adult subjects, this report is consistent with other studies showing high efficacy and no serious adverse events against either *P. falciparum* or *P. vivax* [21]. DHA/PPQ with its longer prophylactic tail has been modelled to be more cost-effective and superior in reducing clinical incidence and malaria prevalence than AL as first-line treatment in higher transmission settings; however, its impact in lower transmission settings like Ethiopia is less clear [45, 46]. Nonetheless, DHA/PPQ is the first line treatment for *P. falciparum* in neighbouring Somalia and Eritrea [21, 24]. The high efficacy for both *P. falciparum* and *P. vivax,* as well as the long half-life, makes DHA/PPQ a good option for treatment and chemoprevention strategies in malaria elimination settings in Ethiopia and elsewhere [25].

Limitations to this current study are several including the lower than targeted enrolment for the DHA/PPQ arms due to enrolment not being extended past the high transmission season. The inclusion criteria for *P. falciparum*-infected patients were reduced to 500 parasites/ul in order to increase enrolment rates; however, only nine patients had parasitaemia levels between 500 and 1000 parasites/ul and likely did not affect the efficacy outcomes greatly. The study only enroled adult patients (18 years and above) for the DHA/PPQ arm which resulted in lower baseline parasitaemia in the DHA/PPQ arms, increased median age for the study overall, lower enrolment and limited generalizability to younger age groups. The study was powered for efficacy outcomes per arm and cannot be used to compare results between arms. In addition, insufficient numbers of patients were enroled in a given health centre necessitating pooling of data from two study sites which resulted in not being able to provide site-specific efficacy estimates. Though sample size was not achieved, site-specific outcomes have been included which showed no major difference from the pooled analysis for the primary outcomes (Additional file 2: Tables S1–S4). Data on the types and prevalence of adverse events was not presented as the study team erroneously recorded ongoing presenting symptoms as adverse events; however, there were no serious adverse events or unusual adverse events e.g. cardiac complaints. Another study limitation was that the second AL dose was unobserved and only confirmed verbally during the next visit which might have resulted in lower adherence in the AL arm. Lastly, the genotype investigation was limited to 50 samples and resistance markers for piperaquine and CQ markers were not included. Studies by Mohammed et al. showed high genetic diversity and multiplicity of infection in samples collected from Northwest and Southwest Ethiopia, reinforcing the need for careful interpretation of genotype results [47–51]. As next steps, consideration should be given to conducting therapeutic efficacy studies with genomic investigations to enable the early detection of resistance, inclusion of age groups below 18 years of age in subsequent studies of DHA/PPQ, powered sample size for between group comparisons and evaluation of other WHO-recommended drugs to expand the anti-malarial arsenal for Ethiopia and beyond.

## Conclusions

This study demonstrated high therapeutic efficacy for the anti-malarial drugs currently used by the malaria control programme in Ethiopia and provides information on the efficacy of DHA/PPQ for the treatment of *P. falciparum* and *P. vivax* as an alternative option. The study reported high efficacy of AL and CQ against uncomplicated *P. falciparum* and P. vivax infections, respectively, over 28 days of follow up. The study provided evidence that the drugs remain efficacious despite decades of use as shown by rapid fever and parasitaemia clearance by day three and the absence of relevant *Pfk13* haplotypes for artemisinin. The high efficacy for DHA/PPQ (100%) over the 42 days of follow up for both *P. falciparum* and *P. vivax* supports the potential use of DHA/PPQ as an additional option for the treatment and chemoprevention of both *P. falciparum* and *P. vivax* in Ethiopia. Further comparisons investigating duration of protection and cost- effectiveness between DHA/PPQ, AL, and CQ in Ethiopia are warranted.

## Declarations

The findings and conclusions in this presentation are those of the authors and do not necessarily represent the official position of the U.S. Centers for Disease Control and Prevention or the U.S. Agency for International Development.

Use of trade names is for identification only and does not imply endorsement by the U.S. Centers for Disease Control and Prevention/the Agency for Toxic Substances and Disease Registry, the U.S. Public Health Service, or the U.S. Department of Health and Human Services.

## Supplementary Information

**Additional file 1.** Microsatellite genotyping for determining recrudescence andreinfection.

**Additional file 2: Table S1**. study profile and characteristics by site. **Table S2**. Treatment outcomes per site. **Table S3**. Proportion of slides negative for asexual parasites on day 2 and 3 per site. **Table S4**. Treatment outcome per site.

## Data Availability

The datasets used and/or analyzed during the current study are available from the corresponding author on reasonable request. Site specific results and genotyping data are included as supplemental information.

## Abbreviations

ACPR: Adequate Clinical and Parasitological Response
ACT: Artemisinin-based Combination Therapy
AL: Artemether–Lumefantrine
CI: Confidence interval
CQ: Chloroquine
DHA/PPQ: Dihydroartemisinin–piperaquine
EPHI: Ethiopian Public Health Institute
ETF: Early treatment failure
FMHACA: Food, Medicine and Health Care Administration and Control Authority of Ethiopia
LCF: Late clinical failure
LPF: Late parasitological failure
MOH: Ministry of health
PCR: Polymerase Chain Reaction
*Pfk13*: *Plasmodium falciparum Kelch 13 gene*
*Pfpmdr1*: *Plasmodium falciparum* Multidrug resistance 1
PMI: U.S. President’s Malaria initiative
PQ: Primaquine
WHO: World Health Organization

## Declarations

### Data availability statement

Data set used is available from the Ethiopian Public Health Institute and/or the curresponding author on modest request.
