# Correction: Therapeutic efficacies of artemether-lumefantrine and dihydroartemisinin-piperaquine for the treatment of uncomplicated Plasmodium falciparum and chloroquine and dihydroartemisinin-piperaquine for uncomplicated Plasmodium vivax infection in Ethiopia

**DOI:** 10.1186/s12936-023-04537-y

**Published:** 2023-04-11

**Authors:** Ashenaf Assefa, Hussein Mohammed, Anjoli Anand, Adugna Abera, Heven Sime, Anna A. Minta, Mekonnen Tadesse, Yehualashet Tadesse, Samuel Girma, Worku Bekele, Kebede Etana, Bereket Hailegiorgis Alemayehu, Hiwot Teka, Dereje Dilu, Mebrahtom Haile, Hiwot Solomon, Leah F. Moriarty, Zhiyong Zhou, Samaly Souza Svigel, Bryan Ezema, Geremew Tasew, Adugna Woyessa, Jimee Hwang, Matthew Murphy

**Affiliations:** 1 Ethiopian Public Health Institute, Addis Ababa, Ethiopia; 2 Institute for Global Health and Infectious Diseases, University of North Carolina at Chapel Hill, Chapel Hill, NC USA; 3 Epidemic Intelligence Service, U.S. Centers for Disease Control and Prevention, Atlanta, GA USA; 4 Malaria Branch, U.S. Centers for Disease Control and Prevention, Atlanta, GA USA; 5 ICAP at Columbia University, Addis Ababa, Ethiopia; 6 U.S. President’s Malaria Initiative, USA Agency for International Development, Addis Ababa, Ethiopia; 7 World Health Organization, Addis Ababa, Ethiopia; 8 Ethiopian Federal Ministry of Health, Addis Ababa, Ethiopia; 9 U.S. President’s Malaria Initiative, Malaria Branch, US Centers for Disease Control and Prevention, Atlanta, GA USA

**Correction: Malaria Journal (2022) 21:359** **https://doi.org/10.1186/s12936-022-04350-z**

Following publication of the original article [1], the authors flagged that they had erroneously reported (in Table 4 + the subsection 'Resistance markers') an S1034N mutation in the Pfmdr1 gene. In addition (in the subsection 'Resistance markers'), they erroneously reported a total of 50 samples in place of a total of 50 Day 0 samples.

The original article has been updated to correct these errors. The authors thank you for reading and apologize for any inconvenience caused.
