# Supplementary material for: Therapeutic efficacies of artemether-lumefantrine and dihydroartemisinin-piperaquine for the treatment of uncomplicated Plasmodium falciparum and chloroquine and dihydroartemisinin-piperaquine for uncomplicated Plasmodium vivax infection in Ethiopia
Source: Malar J. 2022 Dec 1;21:359. doi: 10.1186/s12936-022-04350-z (PMC9714156; doi:10.1186/s12936-022-04350-z)
Supplement: Supplementary file 2 — Additional file 2: Table S1. study profile and characteristics by site. Table S2. Treatment outcomes per site. Table S3. Proportion of slides negative for asexual parasites on day 2 and 3 per site. Table S4. Treatment outcome per site. [file 12936_2022_4350_MOESM2_ESM.docx]

|  | Felgeselam (Pawe) | | | | Arbaminch | | | |
| --- | --- | --- | --- | --- | --- | --- | --- | --- |
|  | Pf | | Pv | | Pf | | Pv | |
|  | AL | DP | CQ | DP | Al | DP | CQ | DP |
| *Enrollment and follow-up* | | | | | | | | |
| Screened, n (Pending) |  |  |  |  |  |  |  |  |
| Enrolled, n | 93 | 63 | 49 | 8 | 13 | 12 | 93 | 48 |
| Lost or withdrew, n (%) | 0 (0) | 3 (5) | 0 (0) | 0 (0) | 1 (8) | 2 (17) | 6 (7) | 6 (13) |
| Excluded, n (%) | 3 (3) | 1 (2) | 6 (12) | 0 (0) | 1 (8) | 1 (8) | 1 (1) | 1 (2) |
| Reached study endpoint, n (%) | 90 (97) | 59 (93) | 43 (88) | 8 (100) | 11 (84) | 9 (75) | 86 (92) | 41 (85) |
| *Participant characteristics at baseline* | | | | | | | | |
| Median age, years (range) | 15  (1-55) | 25  (18-65) | 12  (5-70) | 27  (18-65) | 24  (10-57) | 23.5  (18-54) | 17  (1-70) | 23  (18-70) |
| Median weight, kg (range) | 42  (8-73) | 52  (39-71) | 30  (13-61) | 56.5  (49-66) | 53.1  (21.1-79.1) | 57.6  (40.9-70.8) | 48.4  (8.9-77.9) | 58.5  (40.8-105.9) |
| Percent female, % | 34.4 | 25.4 | 40.8 | 37.5 | 30.8 | 50.0 | 45.2 | 35.4 |
| Median day 0 parasitemia, parasites/µl (range) | 22834  (621-97326) | 8246  (524-85321) | 10240  (882-34846) | 4833  (737-28080) | 12000  (520-31200) | 12200  (2480-96000) | 9880  (520-48320) | 5460  (800-30320) |
| Median day 0 gametocyte density, gametocytes/µl (range) | 0  (0) | 0  (0–3520) | 287  (0–8400) | 167  (33–880) | 0  (0) | 0  (0) | 551  (0–14023) | 340  (0–10000) |
| Median day 0 hemoglobin, g/dl (range) | 13.2  (8.6-17.3) | 13.3  (7.4-16.4) | 12.4  (10.2-15.4) | 14.1  (12.4-15.7) | 14.5  (12.1-18.7) | 14.6  (10.7-17.6) | 13.2  (7.3-18.3) | 14.4  (9.9-17.6) |

**Supplement Table 1. study profile and characteristics by site**

AL: artemether-lumefantrine; DP: dihydroartemisinin-piperaquine; CQ: Chloroquine

Notes:

1. Combined Lost to follow up and Voluntary withdrawals

2. Excluded = protocol violations and subjects removed due to interference

**Supplement Table 2. Treatment outcomes** **per site :** total Treatment failure, ETF, LTF, Day of failure (7, 14, 21, 28, 35, 42), ACPR

|  | n (%) | | | | | | | |
| --- | --- | --- | --- | --- | --- | --- | --- | --- |
|  | Felgeselam (Pawe) | | | | Arbaminch | | | |
|  | Pf | | Pv | | Pf | | Pv | |
|  | AL  *n=90* | DP  *n=59* | CQ  *n=43* | DP  *n=8* | AL  *n=11* | DP  *n=9* | CQ  *n=86* | DP  *n=41* |
| Treatment failure | 2 (2.2) | 0 | 1(2.3) | 0 | 0 | 0 | 2 (2.3) | 0 |
| Early treatment failure | 0 | 0 | 0 | 0 | 0 | 0 | 0 | 0 |
| Late clinical failure | 0 | 0 | 0 | 0 | 0 | 0 | 0 | 0 |
| Late parasitological failure | 2 | 0 | 1 | 0 | 0 | 0 | 2 | 0 |
| Day of failure, 21* | 1 | 0 | 0 | 0 | 0 | 0 | 1 | 0 |
| Day of failure, 28 | 1 | 0 | 1 | 0 | 0 | 0 | 1 | 0 |
| Day of failure, 35 | - | 0 | - | 0 | - | 0 | - | 0 |
| Day of failure, 42 | - | 0 | - | 0 | - | 0 | - | 0 |
| Adequate clinical and parasitological response | 88 (97.8) | 59 (100) | 37 (86.0) | 8 (100) | 11 (100) | 9 (100) | 84 (97.7) | 41 (100) |

AL: artemether-lumefantrine; DP: dihydroartemisinin-piperaquine; CQ: Chloroquine

**^*^** Includes day of failure 19

**Supplement Table 3. Proportion of slides negative for asexual parasites on day 2 and 3 per site**

|  | Proportion Slides Negative (95% Confidence Intervals) | | | | | | | |
| --- | --- | --- | --- | --- | --- | --- | --- | --- |
|  | Felgeselam (Pawe) | | | | Arbaminch | | | |
|  | Pf | | Pv | | Pf | | Pv | |
|  | AL | DP | CQ | DP | AL | DP | CQ | DP |
| Day 2 | 94  (88–98) | 100  (94–100) | 98  (89–100) | 100  (63–100) | 100  (74–100) | 100  (72–100) | 100  (96–100) | 100  (93–100) |
| Day 3 | 98  (92–100) | 100  (94–100) | 100  (92–100) | 100  (63–100) | 100  (74–100) | 100  (72–100) | 100  (96–100) | 100  (93–100) |

AL: artemether-lumefantrine; DP: dihydroartemisinin-piperaquine; CQ: Chloroquine

**Supplement Table 4. Treatment outcome per site**

|  | Efficacy (95% Confidence Intervals) | | | | | | | |
| --- | --- | --- | --- | --- | --- | --- | --- | --- |
|  | Felgeselam (Pawe) | | | | Arbaminch | | | |
|  | Pf | | Pv | | Pf | | Pv | |
|  | AL | DP | CQ | DP | AL | DP | CQ | DP |
| Uncorrected | | | | | | | | |
| Per-protocol Day 28 | 98  (92–100) | 100  (94–100) | 98  (88–100) | 100  (63–100) | 100  (73–100) | 100  (69–100) | 98  (92–100) | 100  (91–100) |
| Per-protocol Day 42 | - | 100  (94–100) | - | 100  (63–100) | - | 100  (69–100) | - | 100  (91–100) |
| Kaplan-Meier estimate Day 28 | 98  (91–99) | 100* | 97  (85–100) | 100* | 100* | 100* | 97  (91–99) | 100* |
| Kaplan-Meier estimate Day 42 | - | 100* | - | 100* | - | 100* | - | 100* |

*No failures in sample, unable to calculate 95% CI

Per-protocol efficacy defined as proportion adequate clinical and parasitological response, Kaplan-Meier estimate calculated from estimate of survival function

AL: artemether-lumefantrine; DP: dihydroartemisinin-piperaquine; CQ: Chloroquine
